# Advancing Diversity in Aging and Alzheimer's Disease Research and Clinical Care: Lessons Learned from Educational and Career Trajectories of Recent Mentorship Program Graduates

**DOI:** 10.1177/00914150241253241

**Published:** 2024-05-27

**Authors:** Sheri Thompson, Dennis Trinidad, Emily Woo, Steven Edland, Becky Marquez

**Affiliations:** 1 UC San Diego Herbert Wertheim School of Public Health and Human Longevity Science, La Jolla, California, USA; 2 Department of Neurosciences & School of Public Health, 8784 UC San Diego, La Jolla, CA, USA

**Keywords:** ADAR, MSTEM, diversity, aging, Alzheimer's disease, research, undergraduate, mentorship, measurement, outcomes

## Abstract

Underrepresented minorities (URMs) are disproportionately affected with aging-related conditions and have inadequate representation in gerontology and geriatrics professions. The Mentorship for Advancing Undergraduate Research on Aging (MADURA) Program aims to increase inclusion of URMs by improving undergraduate retention and success, increasing rates of graduate/medical school applications, and increasing entry into aging research/clinical employment. MADURA provides cohorts with faculty and peer mentorship, research skills training, paid research lab experiences and professional development opportunities. About 87% of the 2023 MADURA cohort intends to take 1+ year after receiving a Bachelor's degree, to prepare for graduate education. Planned activities include gaining work experience, preparing for standardized tests, and obtaining formal training to strengthen graduate/medical school applications. In addition to immediate graduate program acceptances, other student outcomes should be assessed. Longitudinal research on the effectiveness of various post-graduation pathways could assist Mentorship programs in supporting their graduates’ longer term educational and career goal attainment.

## Background

*Mentorship for Careers in Aging/Alzheimer's Disease Research and Clinical Care*. Although ethnic minorities in the U.S., including Hispanics/Latinos and Blacks/African Americans, are disproportionately affected with aging-related conditions such as Alzheimer's Disease and dementia (Kornblith et al. 2022; Weiss et al., 2023), they are underrepresented in the professions of aging-related research and clinical care (Harawa et al., 2017). Given that the majority of the U.S. population is projected to be non-White by 2060 (Vespa et al., 2020), addressing the disparities in aging-related disease and mortality is vital. A key step toward reducing disparities is to improve inclusion of students from communities of color in training as aging-related researchers and health care practitioners.

However, the research literature contains few examples of scientifically evaluated programs for underrepresented minority (URM) undergraduates, which are focused on aging-related fields (Ureña et al., 2020). Many undergraduate student programs have aimed to generally increase diversity in STEM and/or health or medical disciplines. These have often focused on providing research experiences, faculty mentoring, and professional development training. Representative goals have included increasing retention of undergraduate students in STEM majors, and increasing application and/or acceptance into STEM graduate school programs. Indeed, higher rates of retention in STEM majors (Lisberg & Woods, 2018), STEM degree graduation rates (Wilson et al. 2012), and intention to pursue STEM-related graduate education (Eagan et al., 2013) have all been demonstrated among undergraduate research program participants, compared to non-participating peers. Interestingly, Latino and Black students are more likely than their White counterparts to report plans to pursue a graduate education, which further highlights the potential benefit of such programs (Eagan et al., 2013).

## MADURA (Mentorship for Advancing Undergraduate Research on Aging)

Based on promising outcomes from general undergraduate STEM and health-focused research training and mentorship programs, the MADURA Program was developed specifically to improve the inclusion of underrepresented minorities in research and clinical care professions related to aging, Alzheimer's Disease and Alzheimer's Disease Related Dementias (AD/ADRD). MADURA is an undergraduate mentorship program funded by the National Institute on Aging and based at the University of California San Diego. It is one of thirty-one currently funded NIA Advancing Diversity in Aging Research (ADAR) R25 educational research programs. Specific Aims are to improve retention and academic success of participating undergraduates, and to increase rates of graduate/medical school applications and/or entry into Aging/ADRD research or clinical employment. MADURA commenced training its first cohort in January of 2021 and is now in its fourth year. Core components are research faculty and peer mentorship, research skills training, paid research lab experiences and professional development opportunities. Trainee cohorts are comprised of 25–35 students per quarter, who come from racial/ethnic backgrounds under-represented in medical or in STEM-related (MSTEM) college majors (UC San Francisco, 2023), and/or who meet at least two NIH Disadvantaged criteria. (USDHHS, 2020). All have STEM or health-related majors and the vast majority are upper division (Junior/Senior) students, with occasional exceptions.

MADURA collects Faculty Mentor and Student Mentee evaluation data for quality assurance, continuous program improvement and outcomes monitoring purposes. Given the relevance to understanding program effectiveness, outcome metrics are of great interest to this ADAR and to other MSTEM mentorship programs. Per the NIA R25 ADAR evaluation criteria (PAR 20–317), program evaluation metrics for undergraduate trainees are to include:

- Aggregate number and demographic characteristics of participants
- Subsequent educational/career progress, including:
  - Successful completion of an undergraduate degree in a STEM field
  - Enrollment in an advanced degree program in a STEM field
  - Continuing engagement with the program through graduation and beyond
  - Evidence of participation in advanced training and research on aging, including applications for research training and research support.

These are certainly face valid and reasonable metrics. However, MADURA faculty mentors and administrators have observed patterns in educational and career progress of our URM and disadvantaged mentees that differ from pathways of majority culture students and those from well-resourced backgrounds. Some important trends are not fully captured solely from the previously listed metrics, or during typical educational program measurement timelines. Mentees are often taking extended (albeit active) breaks before graduate education applications. Even Bachelor's degree completion may take longer for URM undergraduates who deem it more feasible to take fewer academic units at a time for academic, financial, or family reasons. Other observed patterns suggest a need to assess additional outcome metrics and extend timelines for *post-graduation* follow-up. Particularly noteworthy is the folly of using immediate applications or acceptances to graduate or medical programs as a *sole* or *terminal* outcome measure, as many underrepresented and disadvantaged trainee graduates opt to postpone graduate or medical degree program applications by one, two or more years. These factors all have implications for budgetary, census and measurement planning.

A recent literature review yielded *no* formal evaluations of the utility of intentional delays or of specific components of post-baccalaureate gap time activities, for future academic or professional success. Still, educational gap periods have gained tremendous popularity among college graduates in recent years. Some students focus on taking a break from academics and gaining additional life experience and/or personal growth, often through travel. Others are structured, career-focused experiences designed to prepare students for the next educational level. The latter structured activities are often referred to as “bridge years” and formal programs may be called “bridge programs.” The University of California (UC) application system allows applicants to enter this kind of “educational preparation program” experience, which may include “counseling, tutoring, research opportunities or special study opportunities, such as study abroad” (Ask Ms. Sun, 2023, Activities and Programs Section). Advice from educational institutions, suggestions from student advisory groups, and even providers of gap time programming abound, with 1–2 year gaps encouraged for many students upon completion of Bachelor's degrees.

This paper is focused on its trainees’ use of educational preparation gaps (“prep time” or “prep years”), which are specifically intended to facilitate future educational goals, but may or may not involve formal courses or structured programs. Prep time is likely to be even more prevalent among graduates from URM or disadvantaged backgrounds, as it allows autonomous focus on the most necessary tasks, and the freedom to engage in them at one's own pace. This is especially important for students facing greater financial barriers, who may simultaneously need to earn income for graduate studies.

Consistent with commonly touted benefits, we have observed that our Program alumni's prep year delays are goal-driven and highly strategic, and merit additional study. Insights into educational and career approaches taken by recent URM graduates, and the rationale for these approaches, *will enable this and other mentorship and training programs to prepare students to optimize post-graduation prep time*, while they are still participating in their undergraduate programs. To expand knowledge of MSTEM mentees’ post-graduation needs, this paper presents data on the MADURA graduates’ near-term employment and educational activities. It also shares longer term professional development activities and plans, which were described at 6 + months after student separation from the Program. The implications for measurement planning, curriculum additions for near-term graduates, and future research needs are discussed.

## Methods

### 2022/2023 MADURA Trainee Demographics

Upon entry, all MADURA Program recruits are matriculated UC San Diego undergraduates with grade point averages of 3.0 + and an expressed desire to train in research related to aging and/or Alzheimer's Disease. Over the 2022–2023 academic year, MAUDRA enrolled and supported 27 undergraduates (21 identifying as female and 6 as male), for each of three academic quarters. Twelve of these cohort members graduated in June 2023, and the remainder will continue for the next academic year. All trainees belonged to an underrepresented racial or ethnic minority in MSTEM higher education, and/or met two or more NIH Disadvantaged criteria. Many students were multi-ethnic but predominantly identified as Hispanic/Latino, with an additional three identifying primarily as Vietnamese, and one as Afghan and one as Native American. Half of the cohort also met 2+ NIH Disadvantaged criteria. Students came from five different educational majors, in conjunction with various concentrations and specialties. Specifically, in 2022/23 MADURA trained 13 biology majors (8 human biology, 3 neurobiology, 1 molecular and cellular biology, and 1 general biology). The cohort also included three general Cognitive Science majors and five Cognitive and Behavioral Neuroscience majors. A Public Health major was represented by five trainees with specializations in global health, medical science, and cognitive science. Lastly, MADURA had one Psychology major.

### Survey Timing and Modality

Immediately upon separation from our program (due to graduation or termination for any reason), all MADURA trainees are asked to complete an Exit Survey, which assesses their experiences in the Program and requests alumni contact information. The Program has been subject to an IRB exception, as data collection serves the purpose of educational program evaluation. However, the Exit Survey also contains an item that presents the option to provide informed consent for follow-up contact and for survey results reporting and publications. To date, only one of 40 MADURA alums has declined follow-up. All others provided phone and email contact information and consented to being contacted by email up to twice annually, for new survey requests. All MADURA surveys are digital, accessed through web links.

At the end of each academic year, the whole cohort completes a series of online surveys. This paper reports results from one: a 2023 whole-cohort survey of Post-graduation Plans that was completed by both imminent graduates and continuing students. Due to growing awareness of gap time use by MADURA alumni, we had also initiated follow up survey administration to Program alums, after Year 2 of implementation. Results of the 2023 Alumni Follow-up Survey are also reported. It should be noted that some of the original consenting MADURA trainees have been lost to follow-up, due to defunct University emails and cell numbers. We now routinely request back-up (non-University) email contact information, to facilitate future follow-up. In order to improve response rates, all alumni were offered a $25 Amazon gift card as an incentive to complete the June 2023 Alumni Follow Up survey.

**Table:** 

Summary of Measures
I. 2023 Whole Cohort Post-graduation Plans Survey - administered to all current trainees at end of the 2022/2023 academic year, excluding two newly enrolled trainees. 23 of the 25 eligible respondents (92%) completed.
II. 2023 Alumni Follow-Up Survey - requests for completion were emailed to all consenting students who had been separated from the Program six months or more; 23 students were contacted and 14 (61%) completed.

## Findings

### I. 2023 Whole Cohort Post-graduation Plans Survey Results

Thirty nine percent (9) continuing and graduating students endorsed receiving employment from mentors or mentor affiliates (beyond the Program), as a *direct result* of their MADURA Program participation. Examples included paid research assistantships or successful referrals to other research job opportunities. As expected from ongoing interactions with trainees, 87% (20) *intended to take one or more gap years after graduation*, and *then* pursue graduate or medical school education. Both continuing trainees and graduates were already pursuing strategic steps toward post-graduation employment and/or graduate education, as outlined in the Table below.

### Highlights:

- In terms of imminent activities (immediately at end of the academic year), 13% (3) had been accepted to graduate programs and another was actively applying.
- None of the June 2023 graduates were accepted to medical school for a Fall start. Only one 2023 graduating senior had already submitted applications; all others with medical school ambitions were planning plan prep time before submitting applications.
- More than half of the students intended to apply to graduate Master's or PhD Programs, while another 43% (10) planned to apply to medical schools.
- 65% (15) were engaged in or planning to do additional academic preparation to strengthen medical/graduate school applications, such as GRE/GMAT preparation courses and study with peers.
- 53% (12) were already saving money toward graduate/medical school education, while another 30% (7) were applying for jobs or plan to do so within 6 months.
- 34% (8) were already actively engaged in or accepted for employment designed to strengthen graduate/medical school applications. Another 13% (3) were currently applying for beneficial work experience, while 39% (9) intended to commence applications within the coming six months (after an initial Summer break).
- Roughly a third were already doing or accepted for short-term Summer internships or training; a few were engaged in or applying for longer term (3 + month) internships or post-baccalaureate training.

### II. 2023 Alumni Follow-Up Survey Results

Although separated from the Program longer than our brand new senior graduates, these 14 respondents were still relatively recent graduates. Only two had reached the two-year post-graduation mark, and half had graduated 6–12 months prior. In terms of currently reported activities, 43% (6) of the alumni had received research-related employment as a *direct result* of MADURA Program participation (e.g., from referrals to colleagues’ research labs or for paid continuation of work in ADAR labs). Five respondents (36%) had now been accepted or were underway in graduate programs, and another was in medical school. Notably, *only two of the surveyed alumni consider their education completed – the other 12 reported future educational aspiration.* Table 2 provides details on current and planned prep time actions.

**Table:** 

*The following is a sample of prep time activities, described by MADURA trainees who intend to pursue graduate education:* “During my gap year I plan to study for the MCAT and work in a clinical setting to save money for med school apps as well as strengthen my experience and connections.” “I am currently in my second gap year gaining more training and experience in research to better prepare myself for grad school.” “Currently, I have a part-time job as a care-giver and study for the MCAT.” “After graduation I took an EMT and Phlebotomy course and got certified for both… I am working on medical school applications, to apply this and possibly next cycle…” “Currently I work as a UX/UI designer for a medical company… I hope to apply for an MPH after two years of saving up.” “I was accepted to the McNair Scholar's Program…” “I am finishing a medical assistant program.” “I applied for a Minority Participation in STEM program…” “I also am volunteering for the UCSD student run free clinic… Also, with the X *(Research and Teaching)* Institute*, I just finished a 5-week immersion course to help at-risk and underserved high school students discover career opportunities in academia. Students worked on both metagenomics and microbiology research.” *

Formal organization name redacted, as a potential source of personally identifying information.

## Discussion

Immediately upon separation, a large majority of students were already engaged in additional Aging/ADRD research or clinical training or employment, *but few had immediately applied to graduate/medical programs*. *A significant majority (87%)* of our 2022/23 cohort (which includes continuing students plus 12 graduates) *intend to take at least one year of prep time, followed by applications for continued education.* Longer-term survey data from MADURA alumni at 6–22 months post-graduation yielded helpful insights about uses of this time. Many of our trainees took, or are in process of taking, time off after BA/BS degree completion, *expressly for the purpose of gaining work experience, preparing for standardized tests (GMAT, GRE…), and obtaining supplemental training to strengthen graduate and medical school applications*. This supports the notion that, in addition to immediate graduate program acceptances, other undergraduate mentorship program outcomes should be assessed, over the longer term. Specifically, relevant job, internship, volunteer and post-bac experiences, standardized test preparation, and financial preparation strategies appear to be prevalent practices and potentially, strong progress indicators. These interim steps may be both necessary and highly strategic, supporting the longer-term success of undergraduate URM MSTEM mentees. However, additional research on the effectiveness of these approaches is also warranted. Research designed to assess the effectiveness of specific prep time activities, and those that may prove counter-productive, would yield helpful information for ADAR and other MSTEM undergraduate mentorship programs.

The MADURA Program results presented here are encouraging. There is a *pervasive shift across domains, from planned trainee activities at the pre-graduation or graduation time point* (Table 1), to *active engagement and acceptances* at *6–22 months post-graduation* (Table 2). Specifically, percentages of students actively engaged in or applying for opportunities in support of advanced education increased at the later time point, for all but one domain. As examples, greater proportions of the longer-separated students reported active participation in saving money for future education; engagement in or applying for resume-building employment, post-baccalaureate or internships; and studying for the GRE/GMAT. Only short-term (≤3 month) internship pursuit was lower among the longer-term alums, presumably because they were now focused on more time-intensive and substantial educational and career goals.

**Table 1.** 2023 Whole Cohort Post-graduation Plans Survey.

Activity *Listed in ascending order, by most prevalent current activity*	Accepted or actively participating Percent (frequency)	Applications or activity in process Percent (frequency)	Plan to apply/start activity within six months Percent (frequency)	Not planned or non-applicable Percent (frequency)
Saving money for additional education	52% (17)	13% (3)	17% (4)	17% (4)
Paid work experience designed to strengthen graduate or medical school applications (not just for income)	35% (8)	13% (3)	39% (9)	13% (3)
Short-term Internships or Summer training (≤3 months)	30% (7)	22% (5)	13% (3)	35% (8)
Studying to improve education program applications (GRE or MCAT prep…)	26% (6)	22% (5)	17% (4)	35% (8)
RESEARCH Work (outside MADURA) in a job related to Aging, Alzheimer's Disease, ADRD, or Neurosciences	26% (6)	9% (2)	35% (8)	30% (7)
CLINICAL or HEALTH SERVICE WORK (outside MADURA) related to Aging, Alzheimer's Disease, ADRD, or Neurology	18% (4)	13 (3)%	30% (7)	39% (9)
Graduate school applications (Masters or PhD)	13% (3)	4% (1)	35% (8)	48% (11)
Longer-term (3+ month) internship programs	4% (1)	13% (3)	14% (4)	65% (15)
Medical School applications	0% (0)	4% (1)	39% (9)	57% (13)
Post-baccalaureate program	0% (0)	13% (3)	17% (4)	70% (16)

From Spring 2023 MADURA Training Cohort Survey of both continuing and graduating trainees (n = 23).

**Table 2.** Use of Prep Time: 2023 “Longer-Term” Alumni Follow-Up Survey.

Activity *Listed in ascending order, by most prevalent current activity*	Accepted &/or actively participating Percent (frequency)	Applications or activity underway Percent (frequency)	Plan to apply/start activity within six months Percent (frequency)	Not planned or non-applicable Percent (frequency)
Saving money for additional education	71% (10)	0% (0)	14% (2)	14% (2)
Paid work experience designed to strengthen graduate or medical school applications (not just for income)	71% (10)	7% (1)	7% (1)	14% (2)
Graduate school applications (Masters or PhD)	36% (5)	0% (0)	14% (2)	50% (7)
RESEARCH Work (outside MADURA) in a job related to Aging, Alzheimer's Disease, ADRD, or Neurosciences	29% (4)	7% (1)	7% (1)	57% (8)
Studying to improve education program applications (GRE or MCAT prep…)	29% (4)	0% (0)	7% (1)	64% (9)
CLINICAL or HEALTH SERVICE WORK (outside MADURA) related to Aging, Alzheimer's Disease, ADRD, or Neurology	29% (4)	0% (0)	14% (2)	57% (8)
Post-baccalaureate program	21% (3)	0% (0)	0% (0)	79% (11)
Short-term Internships or Summer training (≤3 months)	14% (2)	14% (2)	0% (0)	71% (10)
Longer-term (3+ month) internship programs	7% (1)	21% (3)	0% (0)	71% (10)
Medical School applications	7% (1)	21% (3)	0% (0)	71% (10)

From Spring 2023 Survey of Alumni who have been separated for 6 + months (n = 14).

There are limitations which must be considered, however. We must be cautious about inferences of individual progress, as the surveys were not individually linked. Thus, we cannot definitively compare progression along the continuum of planning to action. Some of these findings may be an artifact of data collection from a slightly different pool of respondents, at each time point. Also, response bias cannot be ruled out. The 61% of alumni who completed follow-up surveys could be more likely to have educational and career accomplishments to report, compared to alumni survey non-responders.

Leveraging better data, mentorship programs can improve support of trainees with MSTEM graduate or medical school ambitions. For example, programs may integrate targeted training curriculum and resource referrals, for the most effective uses of post-graduation prep time. Focus should be placed upon those shown to be predictive of medical and graduate program acceptances, or attainment of career goals. Resources allowing, undergraduate mentorship programs would do well to increase follow-up data collection well beyond student graduations, to ensure that data collection is tagged to individual respondents for longitudinal comparisons, that it includes a wide range of interim prep time strategies. Programs are also encouraged to advance the state of MSTEM educational mentorship scientific knowledge, by reporting findings on post-graduation educational and career pathways of their alums.

As previously mentioned, mentorship programs and their trainees also stand to benefit from identifying prep time activities that are negatively associated with graduate/medical program acceptances and attainment of career goals. Ineffective strategies may be avoided, in many cases. However, it should be acknowledged that many mentorship programs may lack the cohort sizes, staff and budget resources to complete longer term follow-up and predictive analyses. Therefore, collaborative, multi-site studies may be a more feasible mechanism for obtaining insights into the most effective gap year strategies for URM mentorship alums’ long-term career success. Such collaborations, which have the potential to expand insights for a broader range of mentee, program and institutional characteristics, are highly recommended.

To summarize, we encourage longitudinal research and dissemination on post-graduation pathways of undergraduate program mentees, since immediate transitions into continuing education are not the only - *nor always the optimal* - road to success. Insights into varied trajectories of successful alumni may better prepare Mentorship programs to partner with their imminent graduates, facilitating implementation of tailored pathways that support longer term educational and career goal attainment.
